# piRNA-like small RNAs mark extended 3’UTRs present in germ and somatic cells

**DOI:** 10.1186/s12864-015-1662-6

**Published:** 2015-06-16

**Authors:** Jennifer Yamtich, Seok-Jin Heo, Joseph Dhahbi, David IK Martin, Dario Boffelli

**Affiliations:** Children’s Hospital Oakland Research Institute, Oakland, CA 94609 USA

**Keywords:** piRNA, 3’ UTR, Somatic, MIWI2

## Abstract

**Background:**

Piwi-interacting RNAs (piRNAs) are a class of small RNAs; distinct types of piRNAs are expressed in the mammalian testis at different stages of development. The function of piRNAs expressed in the adult testis is not well established. We conducted a detailed characterization of piRNAs aligning at or near the 3’ UTRs of protein-coding genes in a deep dataset of small RNAs from adult mouse testis.

**Results:**

We identified 2710 piRNA clusters associated with 3’ UTRs, including 1600 that overlapped genes not previously associated with piRNAs. 35 % of the clusters extend beyond the annotated transcript; we find that these clusters correspond to, and are likely derived from, novel polyadenylated mRNA isoforms that contain previously unannotated extended 3’UTRs. Extended 3’ UTRs, and small RNAs derived from them, are also present in somatic tissues; a subset of these somatic 3’UTR small RNA clusters are absent in mice lacking MIWI2, indicating a role for MIWI2 in the metabolism of somatic small RNAs.

**Conclusions:**

The finding that piRNAs are processed from extended 3’ UTRs suggests a role for piRNAs in the remodeling of 3’ UTRs. The presence of both clusters and extended 3’UTRs in somatic cells, with evidence for involvement of MIWI2, indicates that this pathway is more broadly distributed than currently appreciated.

**Electronic supplementary material:**

The online version of this article (doi:10.1186/s12864-015-1662-6) contains supplementary material, which is available to authorized users.

## Background

Among the numerous types of small RNAs that have been investigated in recent years, piRNAs have been of interest primarily because they seem to be essential for maintenance of germ cells. They range in size from 24–32 nt, complex with members of the PIWI clade of Argonaute proteins, and are enriched for a 5’ uridine [1–6]. piRNAs are present in a broad spectrum of metazoan species, and in the mouse they associate with three Piwi family proteins: MILI, MIWI, and MIWI2 [1–3, 7, 8]. MIWI2 is expressed at high levels in the prepachytene phase of spermatogenesis, where it complexes with piRNAs derived mostly from retrotransposon sequences and is involved in epigenetic suppression of retrotransposon activity [7, 9]. MIWI is expressed in the pachytene stage of meiosis and is required for spermatogenesis. MIWI-associated piRNAs are of a different type and have been implicated in regulation of mRNA and retrotransposon transcripts in differentiating spermatocytes [10–12], but may have other functions as well. When pachytene piRNA sequences are aligned to the genome, they tend to cluster into large regions, reflecting their derivation from longer single-stranded primary RNA pol II transcripts [1, 2, 4, 5, 13]. These piRNA clusters are found at syntenic regions in mouse, rat, and human [2], although orthologous regions in mouse and human produce piRNAs with different sequences [1].

Transposon-derived piRNAs are produced via a ping-pong amplification loop in which primary piRNAs antisense to transposon mRNA sequences direct the cleavage of the transposon mRNA, leading to the production of secondary sense piRNAs which then direct the cleavage of the complementary sequence from the primary piRNA transcript. This results in sense/antisense piRNAs characterized by a 10-bp 5’ overlap, and secondary piRNAs with an A at position ten [5]. In prepachytene mouse testis, these sense/antisense piRNAs tend to be distributed along the length of a piRNA cluster [7].

Less is known about the functions of pachytene piRNAs. They are derived from single-stranded precursors, are not enriched for transposable element sequences, and do not show evidence of ping-pong amplification, suggesting that they are not involved in transposon silencing [13, 14]. They cofractionate with polysomes, and so may be involved in translational control of gene expression [3]. A subset of pachytene piRNA clusters are associated with spliced mRNAs, particularly their 3’ untranslated regions (3’ UTRs) [15–17]. Although it is unclear how certain 3’ UTRs are selected for piRNA production, selection is not based simply on mRNA levels or the expression of specific Piwi proteins [16]. Genes from which piRNAs are derived have more isoforms and antisense transcripts, suggesting a connection between piRNA production, antisense transcription, and alternative splicing [17]. However, it is still not clear if the 3’ UTR is processed into piRNAs, or if they are instead derived from an independent transcript. To address this question, we conducted a detailed analysis of 3’ UTR piRNAs in adult mouse testis, and from somatic tissues. A deep dataset of small RNAs from adult mouse testis identifies many new 3’ UTR piRNA clusters, some of which are also present in somatic cells and are derived from rare and previously unannotated extended 3’ UTRs. Analysis of somatic piRNAs from MIWI2 null mice demonstrates that a subset of 3’UTR clusters is dependent on MIWI2, an unexpected finding that supports a somatic role for MIWI2. Additionally, we found small regions of sequence homology between intergenic and 3’ UTR piRNA clusters, suggesting a mechanism by which intergenic piRNA production could select 3’ UTRs of specific genes for processing.

## Results

### Deep sequencing of small RNAs in the adult mouse testis

We sequenced small RNAs from the adult mouse testis to a depth of approximately 139 million reads. Adapter stripping, removal of known ncRNAs (rRNA, tRNA, snRNA, snoRNA, and miRNA) and collapsing of identical reads left approximately 13 million distinct small RNA sequences (Additional file 1: Table S1). We compared our small RNA sequences to six piRNA datasets, available at the time of analysis, from the adult mouse testis: pachytene spermatocytes, round spermatids and type A spermatogonia [17], MILI- and MIWI-immunoprecipitated piRNAs and total small RNAs [16]. Of the stage-specific small RNAs, our small RNA sequences were most similar to small RNAs from round spermatids and pachytene spermatocytes, capturing 48 % and 40 % of the previously identified unique sequences, respectively (Table 1). Our dataset captured 20 % and 22 % of confirmed piRNA sequences, identified as binding to MILI and MIWI (Table 1). However, previously identified piRNA sequences represented at most 5 % of our RNA sequences, suggesting that we have identified many new and less common small RNA sequences.

**Table 1:** Comparison of small RNA sequences from adult mouse testis

Published dataset	RNA sequences	Shared sequences
Type A spermatogonia (GSM610965)	1,192,011	52,079 (4 %)
Pachytene spermatocytes (GSM610966)	941,683	378,724 (40 %)
Round spermatids (GSM610967)	876,411	419,213 (48 %)
MILI IP (GSM475280)	3,156,297	640,898 (20 %)
MIWI IP (GSM475279)	2,798,127	623,800 (22 %)
Total RNA (GSM475281)	2,742,329	600,957 (22 %)

**Fig. 1:**
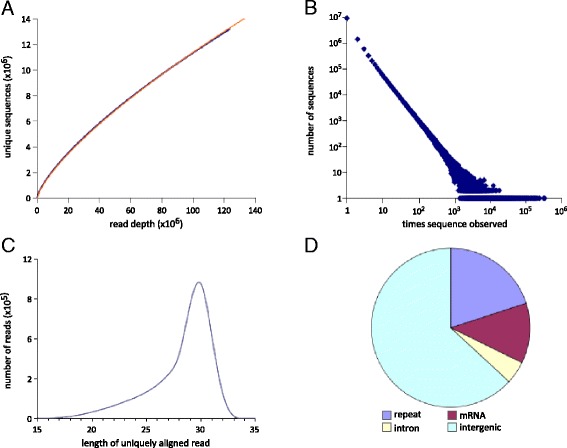
Deep sequencing of adult testis small RNAs does not capture the full complexity of piRNA-like species. Even at extreme read depths, many new unique alignments are identified. **a** The number of observed unique sequences increases as a power function of read depth in our dataset (black line: experimental data; grey line: logarithmic fit). The curve does not approach saturation even at read depth >1.2x10^8^. **b** Frequency with which sequences are observed in the dataset. Most are observed only once or a few times. **c** Length distribution of uniquely aligned small RNAs in adult testis, with duplicate reads removed. There is a distinct peak at 29 nt, with a distribution skewed toward smaller sizes. This size distribution is typical of adult testis piRNAs. **d** Genomic annotation of uniquely aligned small RNAs in testis. The concentration in intergenic regions is typical of piRNAs from the adult testis. Repeat: 20 %; mRNA: 12 %; intron: 5 %; intergenic: 63 %

Despite the increased sequencing depth, our dataset is not saturated. The number of unique sequences is still increasing as a power function of read depth (r = .999, Fig. 1a), and 72 % of the reads in our dataset are for sequences observed only once (Fig. 1b). This lack of saturation likely explains why we found at best only about half of the unique sequences from previously published datasets (Table 1), and is consistent with previous findings that coverage of piRNA datasets is incomplete [17].

We next aligned our unique small RNA sequences to the mouse genome allowing up to one mismatch (Additional file 1: Table S1). Approximately 7.5 million sequences aligned uniquely to the genome (88 % of aligned reads, Additional file 1: Table S1). These small RNA sequences had characteristics typical of piRNAs, such as a length distribution with a single peak centered at 29 nt (Fig. 1c) and a 5’ uridine bias (68 %). They aligned primarily in non-repetitive intergenic regions (63 %, Fig. 1d) corresponding to previously described piRNA clusters [13], with the remaining sequences aligning to repetitive and genic (mRNA and intron) regions (20 % and 17 %, respectively, Fig. 1d). Of the genic sequences, most aligned to regions corresponding to processed mRNA (Fig. 1d). Due to the overall similarity with published piRNA datasets, the piRNA-like sequence length, and the 5’ uridine bias, it is likely we have identified many new murine pachytene piRNA sequences.

### A subset of piRNAs align in clusters at 3’ UTRs

**Fig. 2:**
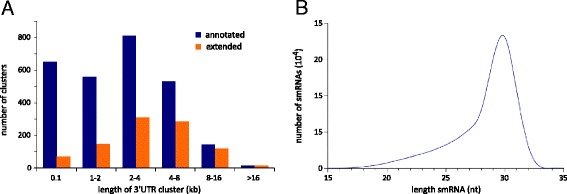
piRNA clusters at 3’ UTRs in adult testis. **a** Length distribution of 3’ UTR piRNA clusters. Blue bars denote clusters of piRNAs aligning to annotated 3’UTRs (RefSeq). Orange bars denote clusters of piRNAs aligning to regions immediately 3’ of annotated 3’UTRs, which are here termed extended 3’ UTRs or xUTRs. **b** Length distribution of piRNAs aligning to 3’ UTR piRNA clusters (both annotated and extended)

Our deep dataset allowed us to identify 2710 clusters of small RNAs that overlapped annotated 3’ UTRs in a sense direction (see Methods; a list of testis piRNA clusters that overlap with 3’ UTRs is in Additional file 2: Table S6). These clusters had a median length of approximately 2.4 kilobases (Fig. 2a, blue bars), and the small RNAs that aligned within these clusters had a length distribution similar to that of the total dataset (Fig. 2b) with an increase in the proportion having a 5’ uridine (72 %). Additionally, we found that these regions were enriched for previously described MILI- and MIWI-associated piRNAs [16] when compared to random non-genic regions of the same size (Additional file 1: Figure S1a), and that the density of small RNAs in our dataset is correlated with the density of confirmed piRNAs in the same regions (r^2^ = 0.78, Additional file 1: Figure S1b). These data suggest that the 3’ UTR clusters we identified are piRNA clusters.

We compared the genes having 3’ UTR piRNA clusters identified in our study with genes identified by reanalysis of previously published piRNA datasets [16, 17]. Our 2710 clusters significantly overlap with clusters obtained from previously reported piRNA datasets, containing 92 % of the 626 3’ UTR piRNA-producing genes identified in the dataset of Robine et al. [16], and 47 % of 1957 identified in the data of Gan et al. [17]. 401 genes were shared by the three datasets, while our dataset contributed an additional 1600 genes. Previous studies identified genes with 3’ UTR piRNA clusters as being involved in nucleic acid metabolism, zinc ion binding, transcription, and regulation-related processes [16, 17]. Our findings are consistent with this, as the top functional categories identified by Ingenuity Pathway Analysis (IPA) are gene expression, post-translational modification, and RNA post-transcriptional modification (Additional file 1: Table S2). Our deeper dataset allowed IPA to identify enrichment for additional categories, including genes involved in infectious disease, the cell cycle, and DNA replication, recombination, and repair (Additional file 1: Table S2).

Under relaxed alignment conditions, 481 clusters (~18 %) contain antisense reads with the 10-bp overlap typical of ping-pong amplification (example in Additional file 1: Figure S2a). This overlap occurred at 1067 sites and involved 76,022 RNA sequences, 13,885 of them antisense to the 3’ UTR cluster. The antisense reads had a decreased 5’ uridine bias (61 %), a bias for an adenine at position 10 (43 %), and were localized in discrete sites generally containing one 10-bp sense/antisense overlap and covering less than 10 % of the cluster region (Additional file 1: Figure S2c-d). The best alignment for 2125 (29 %) of these reads was antisense to the same 3’ UTR sites (Additional file 1: Figure S2b). The remaining 5170 reads (71 %) aligned optimally to piRNA clusters distinct from the 3’ UTR clusters where they were discovered, with 5054 sequences aligning to intergenic piRNA clusters (Additional file 1: Figure S2b). This suggests the possibility of an interaction between 3’UTRs and intergenic piRNA clusters that have regions of sequence similarity, although the rarity of these RNAs does not support any extensive role for such a mechanism.

### piRNA clusters mark extended 3’UTRs

**Fig. 3:**
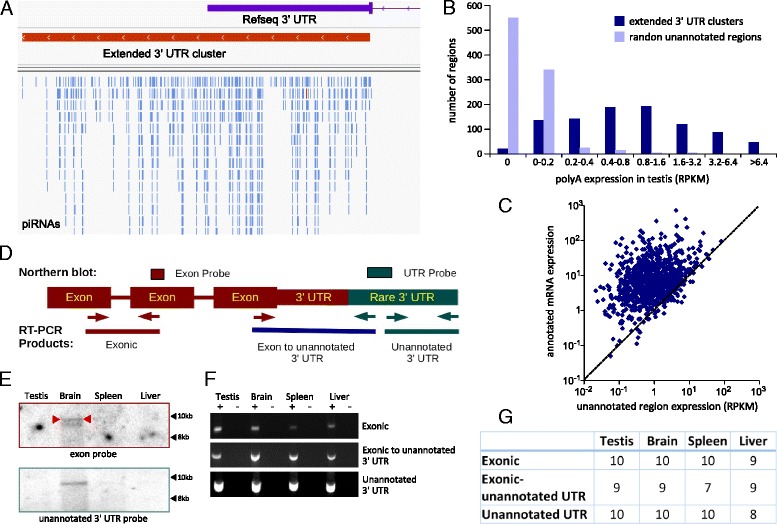
piRNA clusters mark extended 3’ UTRs. Potential precursors of extended 3’ UTR piRNA clusters are detectable as 3’ UTR isoforms in testis and in somatic tissues. **a** Representative example of a piRNA cluster that extends beyond an annotated 3’ UTR. The annotated 3’UTR is shown in purple and the piRNA cluster is shown in red. Individual piRNA alignments are in blue (sense strand) and red (antisense strand) at the bottom. Genomic features are visualized using the Integrative Genomics Viewer [51]. **b** Unannotated portions of 3’ UTR piRNA clusters are enriched for polyA+ RNA expression when compared to randomly selected unannotated regions. We used an adult testis RNA-Seq dataset [18] to determine expression of the unannotated portions of the extended 3’ UTR clusters (dark blue) or random intergenic regions of equal size (light blue). This indicates that the unannotated extended 3’UTRs are transcribed in the testis. **c** Expression of the unannotated portion of extended 3’ UTR clusters (X-axis) compared with expression of the corresponding annotated mRNA (Y-axis) in the adult testis RNA-Seq dataset [18]. The annotated portions of genes are generally expressed at higher RPKM than the unannotated portion of the 3’UTR. **d** Schematic of Northern blot probes, RT-PCR primers, and PCR amplicons. **e** Northern blot of *Pdpr* with an exonic probe (top) shows bands consistent with the length of the annotated mRNA, plus a band consistent with addition of the unannotated region corresponding to the piRNA cluster. The bands are visible only in brain. Probing of the same blot with the unannotated 3’UTR probe (bottom) shows a single band in the same position as the upper band in the exonic probe blot. **f** Representative RT-PCR results of the three *Pdpr* amplicons shown schematically in D and marked to the right, with (+) and without (−) reverse transcriptase during the cDNA synthesis step (top). **g** Summary of RT-PCR products obtained from 10 transcripts with extended 3’ UTR piRNA clusters. For each gene testis, brain, spleen, and liver were tested. All transcripts show evidence of an extended 3’UTR in testis and most also have the extended 3’ UTR in the other tissues as well

Some 3’ UTR piRNA clusters extend past the annotated 3’ UTR (for example, see Fig. 3a). If the 3’ UTR is the precursor that is processed to produce the piRNAs, then piRNAs should not align to regions beyond the 3’ UTR; yet in some cases the greatest read depth lies 3’ of the annotated transcript. We identified 939 3’ UTR clusters that extended beyond the annotated transcript by more than 30 % of the length of the cluster. These clusters were up to approximately 50 kilobases (kb) in length, with a median length of approximately 3.6 kb (Fig. 2a, orange).

The putative extended 3’ UTR regions are unannotated in the gene annotation (refSeq) used for the analysis. However by using an RNA-Seq dataset from adult mouse testis [18] we were able to detect significant expression of 781 (83 %) of these regions (Fig. 3b, expression >0.2 RPKM, which is greater than expression from 95 % of randomly selected regions). Most (95 %) of the unannotated portions of the piRNA cluster precursors were expressed at a lower level than the corresponding annotated mRNA (Fig. 3c). This finding is consistent with the hypothesis that these piRNA precursors are rare 3’ UTR isoforms rather than independently transcribed piRNA precursors.

Since RNA-Seq cannot demonstrate that reads from the extended 3’UTR clusters are derived from the same transcript as the annotated mRNA, we analyzed one of the highly expressed unannotated 3’ UTR piRNA precursors (*Pdpr*) by Northern blotting of testis RNA (Fig. 3d); we also tested three somatic tissues because it has been suggested that piRNAs may be expressed in somatic tissues [19]. Northern blotting detected two brain transcripts, with lengths corresponding to the annotated mRNA and to the annotated mRNA plus the extended 3’ UTR (Fig. 3e, exon probe panel). We also carried out RT-PCR on 10 transcripts associated with piRNA clusters extending beyond the annotated 3’UTR. In 9 of the 10, RT-PCR detected transcripts extending from the annotated portion of the mRNA to the unannotated extended 3’UTR (an example is shown in Fig. 3f; the summary for all PCRs is in Fig. 3g). Taken together, these data suggest that many piRNAs aligning to regions beyond annotated 3’ UTRs are processed from polyadenylated 3’ UTRs that are not present in current genome annotations, i.e., the presence of an extended 3’UTR cluster predicts the existence of a longer alternative 3’UTR. For convenience, henceforth we will refer to these UTRs as testis xUTRs (extended UTRs).

### xUTRs in somatic tissues

Because we noted evidence of 3’ extended transcripts in brain (Fig. 3e-g), we sought evidence of xUTRs in somatic RNA-Seq datasets from the ENCODE project. This analysis strongly supports the expression of xUTRs in a variety of tissues (Additional file 1: Figure S3). Most (79-93 %, depending on the tissue) testis xUTRs are also present in somatic tissues; the unannotated regions have median expression levels 0.18-0.33 fold that of the annotated expressed mRNA (Additional file 1: Figure S3). The highest expression of xUTRs was observed in cerebellum (Additional file 1: Figure S3). These RNA-Seq findings are consistent with results of RT-PCR, which detects transcripts extending from annotated portions of the mRNA to the unannotated piRNA precursor in brain, spleen, and liver (Fig. 3f-g). This suggests that precursor transcripts for the xUTRs are polyadenylated, less abundant than the canonical transcript, and in most cases not germline-specific.

**Fig. 4:**
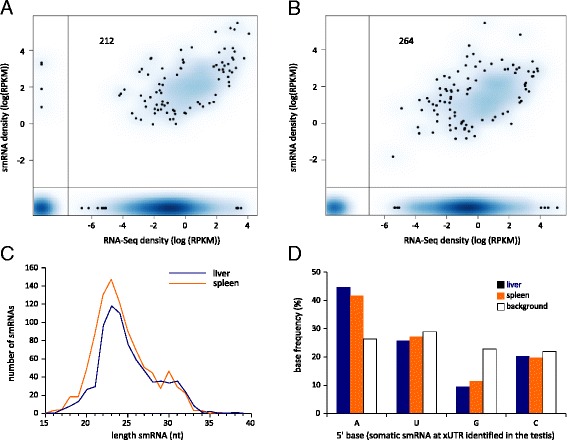
Somatic small RNAs align to xUTRs defined in the testis. **a** and **b**. Density scatterplots of poly-A mRNA expression (x-axis, data from ENCODE) versus expression of novel small RNAs (y-axis, from this project) at genes with xUTRs. The intensity of the blue color is proportional to the number of genes with a given level of poly-A mRNA and small RNA expression, measured as log (RPKM); dots in areas of lowest regional density represent individual genes. Plots are generated with smoothScatter in R. 212 testis xUTRs have both mRNA and smRNA reads in liver (**a**), and 264 in spleen (**b**); read densities of smRNA and mRNA at these clusters are positively correlated (top right quadrant). Many testis xUTRs have mRNA reads in these tissues but lack smRNAs (bottom right quadrant). **c** Somatic small RNAs aligning to testis xUTRs are not piRNA-like. Length distribution of somatic small RNAs aligning to testis xUTRs in liver (black line) and spleen (grey line): the peak at 23 nt indicates that somatic smRNAs are shorter than adult testis piRNAs. **d** Novel small RNAs aligning to testis xUTRs are enriched for 5’ A in liver and spleen. Background is the base frequency across these unannotated 3’ UTR regions

Since we find evidence of xUTRs in somatic tissues, we asked if small RNAs aligning to xUTRs are present in somatic tissues. To address this question we sequenced small RNAs from mouse liver and spleen. After processing the sequence data and removing sequences matching known noncoding RNAs, we were left with approximately 328,000 and 410,000 unique small RNA sequences in liver and spleen, respectively (Additional file 1: Table S3). We then identified small RNAs mapping to the testis xUTRs, and compared them with the RNA-Seq datasets from liver and spleen (Fig. 4. a: liver; b: spleen). This strategy identified three categories of somatic 3’UTR, relative to the xUTRs defined in the testis. In the first group, neither the xUTR nor small RNAs derived from it were expressed in liver or spleen (138 (15 %) liver and 102 (11 %) spleen, bottom left quadrants in Fig. 4a-b). In a second group, small RNA density was proportional to RNA-Seq density at the xUTR (top right quadrants in Fig. 4a-b, r_liver_ = 0.53, r_spleen_ = 0.42). Finally, in many cases the xUTR was present but no small RNAs were detected (585 (62 %) liver and 573 (61 %) spleen, bottom right quadrants in Fig. 4a-b). In contrast to the testis, small RNAs aligning to somatic xUTRs are not piRNA-like: their length distribution peaks around 23 nt (Fig. 4c), and they have a preference for 5’-A (Fig. 4d). The presence of these small RNAs is not merely a consequence of high levels of expression, since many highly expressed genes lack somatic xUTR small RNA clusters (Fig. 4a-b).

Our identification of somatic 3’UTR-associated small RNA clusters led us to ask whether there are tissue-specific somatic 3’UTR piRNA clusters that are not found in the testis (see Methods for details). We identified 488 genes enriched for 3’ UTR small RNAs in the liver, and 345 in the spleen. Similar to the somatic small RNAs aligning to xUTR defined in the testis (above), small RNAs in these somatic clusters were not piRNA-like, but had a peak length distribution of 23 nt (Additional file 1: Figure S4a) and enrichment of A and U at the 5’ base (Additional file 1: Figure S4b). Many highly expressed genes in liver and spleen lacked these RNAs, suggesting that small RNAs identified in these clusters are not likely to be merely degradation products of highly expressed genes (Additional file 1: Figures S4c, d). GO term analysis indicates that most genes from each of the three tissues are associated with terms that are found only for that tissue, i.e., each tissue has a distinct set of functions that is associated with extended 3’UTRs and small RNAs (Additional file 1: Figure S4e and Additional Table S4).

### A subset of somatic 3’UTR small RNA clusters are MIWI2-dependent

**Fig. 5:**
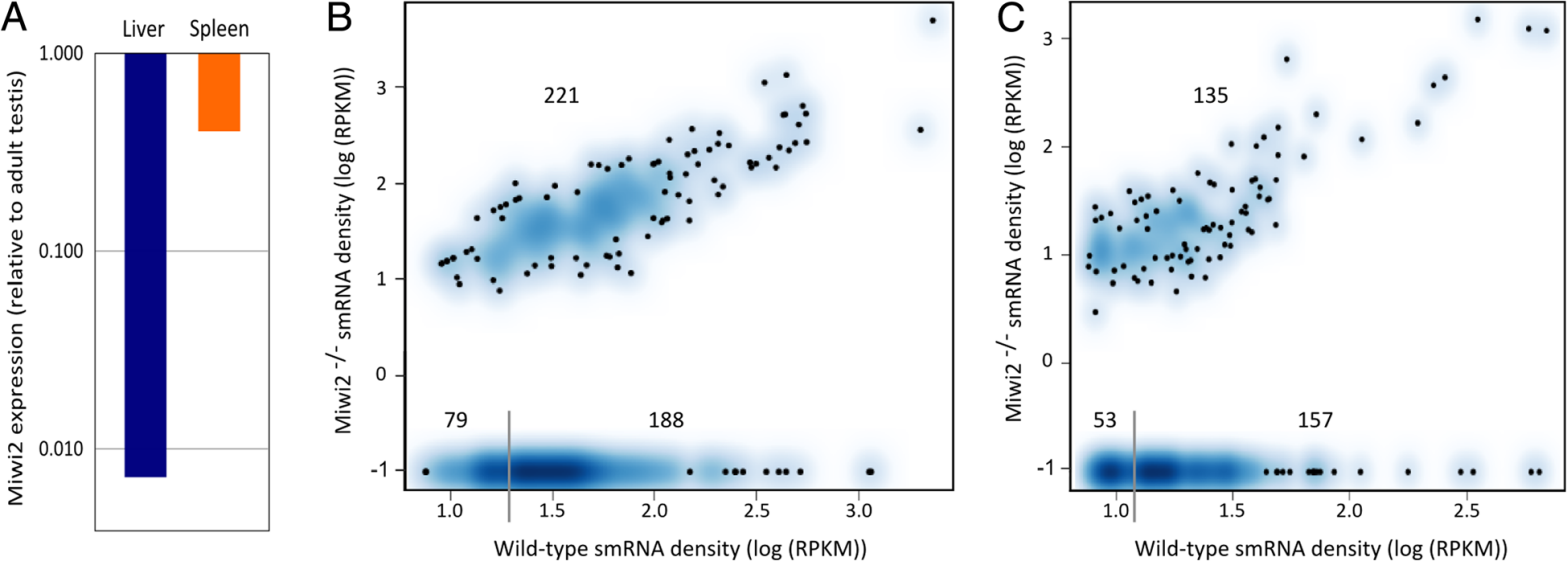
Dependence on MIWI2 defines two classes of somatic 3’ UTR small RNA clusters. **a** Expression of Miwi2 in mouse spleen and liver, assayed by quantitative RT-PCR. Results are expressed relative to the level in adult mouse testis; expression is reported on a logarithmic scale, and shows the degree to which it is lower than expression in testis. B and C) Density scatterplots of expression of small RNAs aligning to 3’UTR clusters in wild type mice (x-axis) and MIWI2^−/−^ mice (y-axis) in liver (**b**) and spleen (**c**). The intensity of the blue color is proportional to the number of genes with a given level of small RNA expression, measured as log (RPKM); dots in areas of lowest regional density represent individual genes. Plots are generated with smoothScatter in R. A subset of 3’ UTR smRNA clusters is absent in MIWI2^−/−^ mice (bottom, to right of gray line). The gray line separates smRNAs expressed at very low levels

In surveying expression of Piwi family members in somatic tissues, we noted expression of MIWI2 (*Piwil4*) mRNA in liver and spleen (Fig. 5a); thus we considered the possibility that MIWI2 participates in the pathway that produces somatic xUTR-derived small RNAs. To test this idea we sequenced small RNAs from the liver and spleen of *Miwi2*^−/−^ mice, whose reported phenotype is limited to male sterility and defects in control of retrotransposons in the testis [7, 9, 20]. Following removal of known ncRNAs, relatively few small RNA sequences mapped to the genome (20,465 and 33,899 in liver and spleen, respectively, Additional file 1: Table S5). Comparison of WT and *Miwi2*^−/−^ somatic xUTR clusters revealed that a set of clusters is absent in *Miwi2*^−/−^ tissues: 267 of the somatic xUTR small RNA clusters present in WT liver, and 210 of those found in the spleen, were absent from these tissues in *Miwi2*^−/−^ mice (Figs. 5b, c). The remaining somatic xUTR small RNA clusters present in WT mice were also present in the *Miwi2*^−/−^ mice.

To compare the properties of the MIWI2-independent and MIWI2-dependent somatic xUTR clusters, we first removed clusters that had small RNA density in the lowest quartile in the WT dataset and no reads in the *Miwi2*^−/−^ dataset (the reads to the left of the grey bar at the bottom of Figs. 5a,b). This filter, which removes the most weakly supported clusters, retained 188 (39 %) and 157 (46 %) MIWI2-dependent 3’ UTR clusters in liver and spleen, respectively (Figs. 5a, b). MIWI2-dependent and independent clusters did not show differences in length distribution, 5’ base composition (Additional file 1: Figures S5a, b), or expression between genes (Additional file 1: Figure S5c, based on RNA-Seq data). Finally, we examined the expression of the coding region and 3’ UTR in a subset of genes with MIWI2 dependent and independent clusters of small RNAs in WT and MIWI2 KO mice. There were no significant and consistent differences in expression in WT and MIWI2 KO liver and spleen between these two groups (Additional file 1: Figure S6). Although we can find no evidence that that knockout affects the expression of genes with MIWI2-dependent somatic xUTRs, the finding that some somatic xUTR clusters are dependent on MIWI2 provides in vivo evidence for a somatic activity of MIWI2.

## Discussion

We analyzed RNAs uniquely aligning to 3’ UTRs in a deep dataset of small RNAs from the adult mouse testis, identifying approximately 1600 new genes with 3’ UTRs that may be piRNA precursors, as well as nearly 1000 clusters with piRNAs that align to previously unannotated extended 3’ UTRs. piRNA-like small RNAs aligning to many 3’UTRs are also present in somatic tissues, where they appear to mark extended 3’UTRs; a subset of these somatic clusters is missing from somatic tissues of *Miwi2*^*−/−*^ mice. This novel evidence that MIWI2 has a role in the somatic production or maintenance of small RNAs presents interesting problems for understanding of its functions. While these studies indicate a possible function for piRNA-like RNAs in modulating 3’UTR length, the significance of this modulation is uncertain, however it may be related to recent evidence that pachytene piRNAs, including the subset derived from the 3’ UTRs of mRNAs, can contribute to degradation of mRNAs and retrotransposon transcripts during spermatogenesis [10–12].

Although our dataset overlapped with 3’ UTR piRNA clusters identified by reanalysis of previously published piRNA datasets [16, 17], due to the general lack of saturation in these datasets there were also many 3’UTR clusters unique to each dataset. By combining the three datasets, we annotated 3771 genes as producing 3’ UTR piRNA clusters in the adult mouse testis. Of these genes, 3721 were eligible for function and pathway analysis with IPA, which identified transcription and modification of protein as the top functions enriched in the combined gene set (Additional file 1: Table S4). These are consistent with the functional categories identified in previous reports [16, 17]. However, by analyzing the combined datasets, certain canonical pathways gained significance. One example is the FLT3 Signaling in Hematopoietic Progenitor Cells pathway (24 of 72 genes in the pathway had 3’ UTR piRNA clusters, *p* = 9.47 e-6), a pathway that stimulates the proliferation of stem cells and progenitor cells [21].

The evidence we have found is consistent with the interpretation that piRNAs and smaller somatic RNAs are derived from extended 3’UTRs, implying that their production may be connected in some way to regulation of 3’UTR length. The 3’ UTR is critical for regulating mRNA stability, localization, and translation (reviewed in [22]). Part of this regulation is influenced by 3’ UTR length, which has been shown to vary with cellular proliferation and reprogramming [23, 24]. Shorter 3’ UTR isoforms are generally expressed in cells with higher proliferation, such as iPS cells, cancer cells, and the testis [24–26], with a shift toward longer 3’ UTR expression as cells differentiate during embryogenesis [24]. mRNAs with these shorter 3’ UTR isoforms have increased stability and protein expression [25], suggesting that shortening of 3’ UTRs in testis, perhaps involving the production of piRNAs, could lead to increased expression of the associated genes. The evidence that extended 3’UTRs, and small RNAs derived from them, are also present in somatic tissues implies that this mechanism may be broadly active, but play a much more prominent role in differentiation of spermatocytes. Recent work has indicated that pachytene testis piRNAs are involved in degradation of retrotransposon transcripts and mRNAs [10–12]; it remains to be established how these findings relate to those described here. We do not posit a causal relationship between the small RNAs we describe and the shortening of 3’UTRs, because our evidence does not directly demonstrate such a relationship.

Perhaps the most intriguing finding of this study is that small RNAs aligning to extended 3’UTRs are also found in somatic tissues, and that a subset of these somatic 3’UTR clusters is absent in mice lacking a functional *Miwi2* (*Piwil4*) gene. The small RNAs aligning to extended 3’UTRs in somatic tissues differ from canonical piRNAs: they are shorter, lack the predominant 5’ uracil, and much less abundant than their testis counterparts. We assessed the role of MIWI2 in these clusters because we and others have found piRNAs in somatic tissues and cancer cells [19, 27], and we find low levels of *Miwi2* mRNA in somatic tissues (Fig. 5a). No phenotype except male sterility has previously been noted in *Miwi2*^*−/−*^ mice [9, 20, 27, 28], although we have found that knockdown of *Miwi2* expression results in differentiation of mouse erythroleukemia (MEL) cells [27]. The absence of somatic xUTR clusters is in effect a phenotype in *Miwi2*^*−/−*^ mice, but any consequences of this absence must be subtle: we find no apparent change in expression of the genes associated with these UTRs in *Miwi2*^*−/−*^ mice, and no other effects have been reported. MIWI2 has been shown to associate with piRNAs in the prenatal testis, and to mediate transcriptional silencing and methylation of retrotransposons at this stage of development [7, 9]. If it is involved in the generation or maintenance of small RNAs derived from 3’UTRs in somatic cells, this would imply some very different function, perhaps related to the recently demonstrated role of MIWI degradation of RNA transcripts [10–12].

## Conclusions

Using a very deep dataset of piRNAs from mature mouse testis, we show that clusters of piRNAs mark extended 3’UTRs that have not been previously annotated. These clusters are present on many mRNAs expressed in the testis, but also in somatic cells. A subset of somatic 3’UTR-derived small RNA clusters are dependent on MIWI2. MIWI2 is a Piwi protein that has been shown to associate with piRNAs in the prenatal testis, and to mediate transcriptional silencing and methylation of retrotransposons at this stage of development. Its potential involvement in the generation or maintenance of small RNAs derived from 3’UTRs in somatic cells would imply some very different function. These findings extend the biological scope of MIWI2 function, and also indicate the existence of a class of extended 3’UTRs that are processed into small RNAs.

## Methods

### Generation and sequencing of small RNA libraries

For testis piRNAs, 10 μg of total RNA from adult mouse testes was electrophoresed on an 18 % denaturing polyacrylamide gel with NEB miRNA markers, the gel stained with SYBR Gold, and the readily visible band migrating at 29 nt (range ~27-31 nt) excised. RNA was eluted from the gel fragment and purified by standard methods. Illumina libraries were constructed from RNA specimens using the Illumina Small RNA Library kit, following the manufacturer’s protocol. Libraries were sequenced on an Illumina GAII apparatus to collect reads of 36 nucleotides. Raw data was processed with the Illumina pipeline v1.3.2. Data are deposited into the GEO database with accession number GSE26251.

For deep sequencing of somatic small RNAs, liver and spleen were harvested from WT and *Miwi2*^*−/−*^ mice [9] and flash frozen in liquid nitrogen (all animal studies were carried out in accordance with CHORI’s Institutional Animal Care and Use Committee regulations). Frozen tissue was homogenized using a Tissue Tearor homogenizer (Biospec Products) and small RNAs (10 – 200 bases) were purified using RNAzol RT (Molecular Research Center, Inc.). Purified RNAs were run on an 18 % denaturing PAGE, the region spanning ~22-35 nt was excised, and RNAs were purified for deep sequencing. RNA libraries were produced as above, with the exception that the Illumina small RNA 3’ adapter v1.5 was used; this adapter is modified by 5’ adenylation. Data are deposited into the GEO database with accession number GSE47093.

### Data processing of small RNAs

Adapter sequences were removed from the 3’ ends of raw reads; up to 25 % mismatch was allowed between the adaptor and read sequences. Exact duplicate sequences were collapsed (only a single representative read was retained). Known contaminants and small RNAs were removed by alignment with Bowtie [29], allowing for up to 3 mismatches. These contaminants and small RNAs included 3’ and 5’ adapter sequences; rRNA (GenBank), tRNA [30–34], and miRNA [35–37] downloaded from the UCSC Genome Browser database [38]; miRNAs from miRBase Release 17 [35, 36, 39, 40]; and snoRNA, snRNA and miRNA downloaded from Ensembl release 65 [41].

### Comparing small RNAs to published datasets

Published small RNA sequences were downloaded from the NCBI Gene Expression Omnibus (GEO) [42], including MILI and MIWI immunoprecipitated and total small RNA from adult mouse testis (GSM475279-GSM475281; [16]) and small RNA sequences from pachytene spermatocytes, round spermatids, and type A spermatogonia (GSM610965-GSM610967;[17]). Similarity indices, the number of sequences in the intersection of the two datasets divided by the number of sequences in the union, were calculated as in [17]. Sequence data from these published sets were reprocessed following the same methods we used to analyze our dataset.

### Small RNA alignment and annotation

Cleaned RNAs were aligned to the NCBI37/mm9 assembly of the mouse genome using Bowtie [29] allowing up to one mismatch and no multiple alignments. Sequences were classified as falling within a genomic feature if at least 50 % of the read length overlapped that feature (BEDTools, [43]). Annotation tracks were downloaded from the UCSC Genome Browser database ([38], November 2010), including those for repetitive elements (RepeatMasker [44]), mRNA (RefSeq exon [45, 46], and introns (RefSeq intron [45, 46]. RNAs were classified as being intergenic if they did not intersect any of the above tracks. Small RNAs that overlapped multiple tracks were classified exclusively in the order of repetitive element > mRNA > intron > intergenic.

### Generation of piRNA clusters

#### Testis dataset

In the testis dataset, we used the feature density estimator F-Seq [47] to identify genomic regions significantly enriched for aligned piRNAs, using a feature length of 1000 and a threshold of 15 standard deviations. To generate 3’ UTR piRNA clusters, we extended the significantly enriched regions by 1 kb if there were 2 piRNA per 1 kb extension and merged overlapping regions. The boundaries of the clusters were then shrunk to the locations of actual aligned piRNAs, and clusters were selected for further analysis if they overlapped a RefSeq 3’ UTR (excluding non-coding genes) by any amount. Unidirectional clusters with over 60 % of reads aligning to a give strand were then selected, and the gene with the greatest 3’ UTR overlap in the sense direction was assigned as the source. The 5’ ends of the clusters were trimmed to match the annotated 3’ UTR, and the boundaries of the clusters were then trimmed to the location of sense piRNAs. If the 3’ end overlapped an adjacent gene, the overlapping portion was removed. However, 3’ UTR clusters that completely overlapped another gene were retained.

To generate other piRNA clusters, we started with the F-Seq defined regions above. We then expanded these regions by 500 bp if there was 1 piRNA in a 500 bp window. As intergenic piRNA clusters are known to span regions with repetitive elements, which would not contain our uniquely aligned reads, we then merged the clusters with the annotated RepeatMasker track elements within 50 bp. We then expanded the clusters by 500 bp windows if they contained 1 piRNA and checked the clusters that had expanded by more than 20 % of their length. We separated clusters if two clusters with reads aligning to different strands had merged. We then shrunk the cluster boundaries to the locations of aligned piRNAs. Clusters were annotated based on the presence of 50 % or more of their contained sequences within a given annotation track (coding exon, UTR, intron). Clusters with fewer than 50 % of sequences aligning to a genic track were classified as intergenic.

#### Somatic datasets

In the liver and spleen small RNA datasets, we identified 3’ UTR clusters by first identifying genes where the density of sense small RNAs in the 3’ UTR was at least two fold that of the density in the coding region. We then selected 3’ UTRs with at least 3 small RNAs and a density of over 7.5 reads per kilobase 3’ UTR per million reads aligned. We set the cluster boundaries as the region of the largest 3’ UTR isoform and extended the 3’ boundary if there was at least 1 sense small RNA per 2 kb.

### 3’ UTR cluster comparison and functional analysis

Genes with 3’ UTR piRNA clusters identified in our testis dataset, Robine et al. [16], and Gan et al. [17], were analyzed with IPA (Ingenuity Systems, www.ingenuity.com). IPA core analysis was conducted using the default parameters. The Functional Analysis identified the biological functions that were most significant to the combined data set. A right-tailed Fisher’s exact test was used to calculate a p-value determining the probability that each biological function assigned to that data set is due to chance alone.

When analyzing the spleen and liver datasets, enrichment of specific gene ontology terms for genes with clusters of small RNAs overlapping their 3’ UTRs was determined using the Functional Annotation tool of the DAVID Bioinformatics Resources [48, 49].

### Antisense reads in 3’ UTR clusters

To analyze antisense reads in 3’ UTR piRNA clusters, we first aligned the small RNA sequences to the genome allowing up to two mismatches and up to 25 alignments. Sites of 10-bp overlap were defined as regions within a 3’ UTR cluster where the first 10 bases of sequences aligned to the plus and minus strands overlapped each other. To determine the most likely genomic source of sequences aligning antisense within the 3’ UTR clusters, these sequences were extracted and re-aligned to the genome under strict conditions (up to one mismatch, unique alignments only). These positions were then annotated based on their presence in an original 3’ UTR cluster, an intergenic piRNA cluster (see above), a different piRNA cluster, or any other location.

### Extended 3’ UTR cluster selection and expression

3’ UTR clusters were selected that extended beyond the annotated 3’ UTR by more than 30 % of the length of the cluster. Clusters that completely overlapped an annotated gene, in the sense or antisense direction, were removed from this analysis. For the expression analysis, only the portion of the cluster that did not overlap the annotated 3’ UTR was considered.

To calculate the expression of the unannotated portion of the extended 3’ UTR cluster precursors, we used mouse testis RNA-Seq data [18] downloaded from the NCBI Sequence Read Archive (ww.ncbi.nlm.nih.gov/sra, accession number SRR036361). We aligned these reads to the mouse genome (assembly NCBI37/mm9) using Bowtie [29] allowing up to one mismatch and no multiple alignments. We used BEDTools [43] to isolate aligned reads that intersected the regions of interest in the sense direction and calculated the expression of these regions by dividing the number of reads in a region by the length of the region and the number of aligned reads and multiplying by 10^9^. We then used BEDTools [43] to generate a set of shuffled intergenic regions of the same size as the unannotated portion of the extended 3’ UTRs, maintaining the same chromosome coverage. Using these regions as background for RNA-Seq expression, we chose a cut-off for positive expression at which 95 % of random intergenic regions are expressed at a lower level.

Expression of the RefSeq mRNA for genes with extended 3’ UTR clusters was calculated as for the annotated portion of the clusters. To calculate expression of the unannotated portion of extended 3’ UTR piRNA precursors in somatic tissues, we first downloaded RNA-Seq data for spleen, lung, liver, kidney, heart, cortex, cerebellum and bone marrow from the ENCODE project (Ren LICR-m group, [50], DCC_Accession wgEncodeEM001706, wgEncodeEM001709-1715). We aligned the sequences and calculated the tissue-specific expression as above.

Gene expression was calculated in spleen and liver in the same fashion. RNA-Seq data for spleen and liver were downloaded from the ENCODE project (Ren LICR-m group, [50], DCC_Accession wgEncodeEM001709 and wgEncodeEM001714, respectively). The density of aligned reads was calculated for mRNA (RefSeq exon [45, 46] as above.

### Northern blot analysis

Mouse testis, brain, spleen and liver were harvested and flash-frozen in liquid nitrogen. Tissues were homogenized in TRIzol Reagent (Invitrogen) using a Tissue Tearor homogenizer (Biospec Products) and total RNA was purified according to the manufacturer’s instructions. mRNA was purified from total RNA using a polyA Spin mRNA Isolation Kit (New England Biolabs) according to manufacturer’s instructions. Northern blotting used the Ambion NorthernMax kit (Invitrogen) following the manufacturer’s instructions. 1 % agarose gels were run with 1 ug mRNA per well and transferred to Ambion BrightStar-Plus membrane (Invitrogen) using downward capillary transfer. RNA was then cross-linked to the membrane using a UV Stratalinker 1800 (Stratagene) and stored at −20 °C. Cross-linked membranes were hybridized to radiolabeled single-stranded DNA probe overnight at 42 °C, washed, and exposed to a phosphorscreen for one week. Phosphorscreens were scanned using a Storm 840 phosphorimager (Molecular Dynamics), and images were analyzed using ImageQuant 5.0.

Template for the Northern blot probe was created by first purifying liver DNA using TRIzol Reagent (Invitrogen) according to the manufacturer’s instructions. Liver DNA was then used as a template for PCR amplification of the probe sequences for *Pdpr*, using primers NblotEx-F and NblotEx-R for the exon-specific probe and NblotUTR-F and NblotUTR-R for the extended 3’ UTR-specific probe (Additional file 3: Table S7). PCR products were purified using a QIAquick PCR purification kit (Qiagen) according to the manufacturer’s instructions.

Primer extension was then used to make radiolabeled single-stranded DNA probes. Radiolabeled probes were purified using SigmaSpin™ Sequencing Reaction Clean-up, Post-reaction Clean-up Columns (Sigma-Aldrich) according to the manufacturer’s instructions.

### RT-PCR of extended 3’ UTR clusters

Total RNA was purified as in the Northern blot analysis, and contaminating genomic DNA was removed with the RNase-Free DNase Set (Qiagen) according to the manufacturer’s instructions. RNA was then cleaned with the RNeasy Mini Kit (Qiagen) RNA cleanup protocol. cDNA was synthesized using the ProtoScript™ M-MuLV Taq RT-PCR Kit (New England BioLabs) according to the manufacturer’s instructions and used as template in the RT-PCR reactions. See Additional file 3: Table S7 for primer sequences, annealing temperatures and extension times. RT-PCR products were run on 1 % agarose gels stained with ethidium bromide and scored as either expressing or not expressing the transcript.

### Some 3’ UTR small RNA clusters are tissue-specific

Since our somatic small RNA datasets were so much smaller than the testis dataset, we used a different approach for identifying 3’ UTRs associated with small RNAs. In the liver and spleen small RNA datasets, we identified 3’ UTR clusters by first identifying RefSeq genes where the density of sense small RNAs in the 3’ UTR was at least two fold that of the density in the coding region. We then selected 3’ UTRs with at least 3 small RNAs and a density of over 7.5 reads per kilobase 3’ UTR per million reads aligned. We set the cluster boundaries as the region of the largest 3’ UTR isoform and extended the 3’ boundary if there was at least 1 sense small RNA per 2 kb.

### Determining MIWI2 dependent and independent somatic clusters

To determine which somatic 3’ UTR smRNA clusters were MIWI2 dependent, we first calculated the density of small RNAs in each 3’ UTR cluster region using datasets from WT and *Miwi2*^*−/−*^ mice as above. If a cluster region had small RNAs in both the WT and *Miwi2*^*−/−*^ datasets, it was determined to be MIWI2 independent. Clusters without any small RNAs in the *Miwi2*^*−/−*^ dataset, and in the bottom quartile of density in the WT dataset, were filtered because they were too small to determine MIWI2 dependence. The remaining clusters, in the top quartiles of small RNA expression in the WT dataset and lacking small RNA alignments from the *Miwi2*^*−/−*^ dataset, were called as MIWI2 dependent clusters.

### qRT-PCR

For quantitative RT-PCR of somatic expression of Miwi2 and of mRNA derived from genes with somatic 3’ UTR small RNA clusters, cDNA was purified as for “RT-PCR of extended 3’ UTR clusters” above. PCR reactions were carried out using FastStart Universal SYBR Green Master (Roche) on an ABI 7900 Real-Time PCR machine according to the manufacturer instructions. All reactions were run in duplicate (quadruplicate for Miwi2 expression), and the mean CT was used for downstream analysis. Expression values were calculated using a standard ladder of pooled cDNA, and normalized for GAPDH expression in that cDNA prep. Values shown are the average and standard deviation obtained from two different mice.

### Availability of supporting data

The data sets supporting the results of this article are available in the NCBI GEO repository, with accession numbers GSE26251 (http://www.ncbi.nlm.nih.gov/geo/query/acc.cgi?acc=GSE26251) and GSE47093 (http://www.ncbi.nlm.nih.gov/geo/query/acc.cgi?acc=GSE47093).

## Additional Files

Additional file 1: Table S1. Data processing for small RNA reads. **Table S2**: Functional enrichment of genes with 3' UTR piRNA clusters. **Table S3:** Data processing for deep sequencing of somatic small RNA. **Table S4.** GO terms associated with tissue-specific xUTR. **Table S5:** Data processing for deep sequencing of somatic small RNA in Miwi2^−/−^ mice. **Figure S1: a)** 3’ UTR clusters in testis are enriched for previously identified piRNAs. **b)** Correlation of the density of piRNAs from our dataset in 3’ UTR clusters with the density of MILI- and MIWI-associated piRNAs at the same 3’UTR clusters. **Figure S2:** Antisense reads in 3’ UTR piRNA clusters show evidence of ping-pong amplification, and are derived from intergenic piRNA clusters. **Figure S3:** Unannotated 3’ UTRs are expressed in somatic tissues. **Figure S4:** Small RNAs aligning to somatic xUTRs. **Figure S5:** MIWI2-dependent small RNAs aligning to somatic xUTRs. **Figure S6:** Expression levels of exons and 3’UTRs from genes with MIWI2-dependent somatic xUTR clusters. Additional file 2: Table S6. List of adult testis piRNA clusters that overlap 3' UTRs. Additional file 3: Table S7. List of PCR primers.
