# Supplementary material for: piRNA-like small RNAs mark extended 3’UTRs present in germ and somatic cells
Source: BMC Genomics. 2015 Jun 16;16(1):462. doi: 10.1186/s12864-015-1662-6 (PMC4469462; doi:10.1186/s12864-015-1662-6)
Supplement: Additional file 1: Table S1. — Data processing for small RNA reads. Table S2: Functional enrichment of genes with 3' UTR piRNA clusters. Table S3: Data processing for deep sequencing of somatic small RNA. Table S4. GO terms associated with tissue-specific xUTR. Table S5: Data processing for deep sequencing of somatic small RNA in Miwi2−/− mice. Figure S1: a) 3’ UTR clusters in testis are enriched for previously identified piRNAs. b) Correlation of the density of piRNAs from our dataset in 3’ UTR clusters with the density of MILI- and MIWI-associated piRNAs at the same 3’UTR clusters. Figure S2: Antisense reads in 3’ UTR piRNA clusters show evidence of ping-pong amplification, and are derived from intergenic piRNA clusters. Figure S3: Unannotated 3’ UTRs are expressed in somatic tissues. Figure S4: Small RNAs aligning to somatic xUTRs. Figure S5: MIWI2-dependent small RNAs aligning to somatic xUTRs. Figure S6: Expression levels of exons and 3’UTRs from genes with MIWI2-dependent somatic xUTR clusters. [file 12864_2015_1662_MOESM1_ESM.pdf]

Additional Material

Yamtich *et al*, “piRNAs mark extended 3’UTRs present in germ and somatic cells”

Additional Tables

Libraries	Reads	Sequences
All	139,338,293	14,858,165
After adapter removal	126,005,581 (90%)	13,346,009 (90%)
After known ncRNA removal	123,437,232 (89%)	13,319,197 (90%)
Mapping to genome (1 mismatch)	112,633,457 (91%)	8,560,560 (64%)
Unique-mappers	104,380,004 (93%)	7,561,049 (88%)
Multi-mappers	8,253,453 (7%)	999,511 (12%)

Additional Table 1: Data processing for small RNA reads

Category	p-Value	# Genes
Gene Expression	1.7E−11	385
Post-Translational Modification	1.3E−09	60
RNA Post-Transcriptional Modification	6.2E−09	81
Infectious Disease	2.9E−08	143
DNA Replication, Recombination, and Repair	8.7E−08	69
Cell Cycle	2.4E−07	264

Additional Table 2: Functional enrichment of genes with 3' UTR piRNA clusters

	Liver		Spleen	
Libraries	Reads	Sequences	Reads	Sequences
All	33,444,464	3,138,340	31,728,186	2,764,166
After adapter removal	29,693,355	2,139,467	28,369,926	2,005,827
After known ncRNA removal	1,400,855	328,464	1,940,299	409,975
Mapping to genome (1 mismatch)	279,748	91,368	442,569	164,019
Unique-mappers	174,904	64,572	273,960	129,176
Multi-mappers	104,844	26,796	168,609	34,843

Additional Table 3: Data processing for deep sequencing of somatic small RNA

Tissue	GO Cluster Annotation	Enrichment
Testis	Membrane-enclosed lumen	17.6
	Zinc ion binding	17.2
	Cellular macromolecule catabolic process	13.7
	Nucleus	10.7
	Chaperone	7.6
Liver	Cofactor metabolic process	5.7
	Vesicular fraction	5.3
	Mitochondrion	4.7
	Acute inflammatory response	3.8
	Lipid binding START domain	3.3
Spleen	Intracellular non-membrane-bound organelle	4.8
	Actin cytoskeleton	2.5
	Ubiquitin associated EF1B, eukaryote	2.4
	Blood vessel development	2.4
	Nucleus	2.2

Additional Table 4. GO terms associated with tissue-specific xUTRs

	Miwi2$^{-/-}$ Liver		*Miwi2*$^{-/-}$ Spleen	
**Libraries**	**Reads**	**Sequences**	**Reads**	**Sequences**
All	5,851,502	381,020	4,880,091	417,232
After adapter removal	5,498,466	270,503	4,248,938	279,836
After known ncRNA removal	405,250	75,887	252,929	83,844
Mapping to genome (1 mismatch)	37,414	20,465	61,637	33,899
Unique-mappers	22,536	13,288	41,644	25,218
Multi-mappers	14,878	7,177	19,993	8,681

Additional Table 5: Data processing for deep sequencing of somatic small RNA in *Miwi2*$^{-/-}$ mice

Additional Table 6: List of adult testis piRNA clusters that overlap with 3' UTRs (separate file in BED format: "Yamtich_SuppTable6_smallRNAclusters_X_3UTR.bed")

Additional Table 7: List of PCR primers (separate spreadsheet: "Yamtich_SuppTable7_primers.xls")

Additional Figures

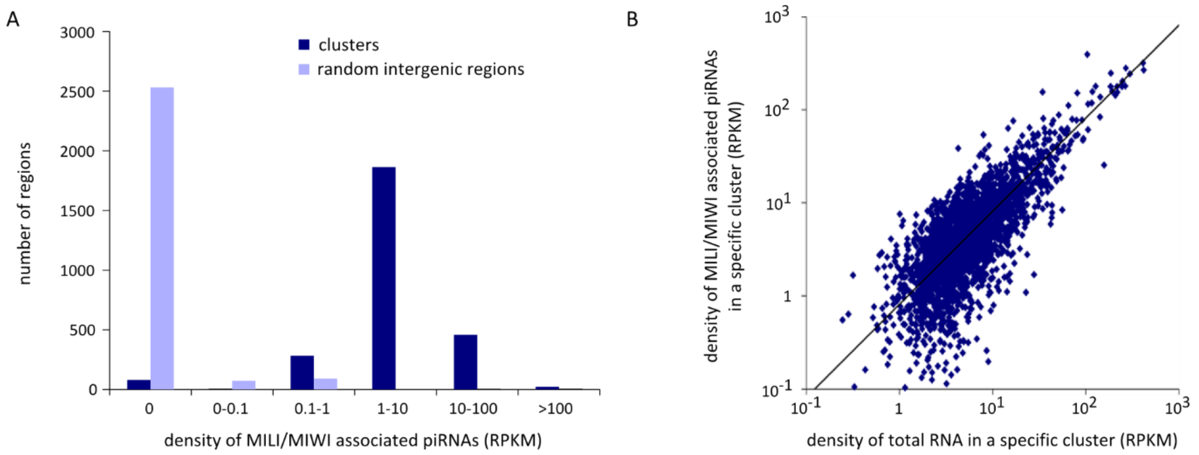

Additional Figure 1: A) 3' UTR clusters in testis are enriched for previously identified piRNAs. The density of previously published piRNAs associated with MILI or MIWI in 3' UTR clusters identified using our testis small RNA data (dark blue) or in random intergenic regions of the same sizes (light blue). piRNAs rarely align to randomly selected regions, but most of our 3'UTR clusters have some piRNAs aligned. **B)** Correlation of the density of piRNAs from our dataset in 3' UTR clusters (x-axis) with the density of MILI- and MIWI-associated piRNAs at the same 3'UTR clusters (y-axis). RPKM, reads per kilobase of cluster per million aligned reads in dataset. Clusters with higher densities of aligned reads from our dataset also have higher densities of aligned reads from MILI and MIWI datasets.

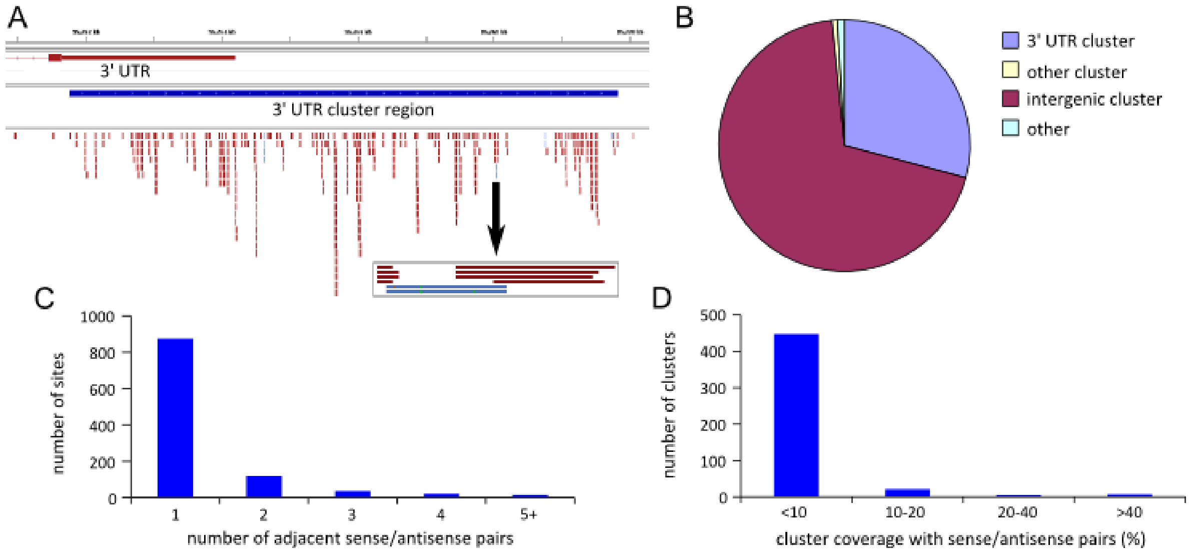

Additional Figure 2: Antisense reads in 3' UTR piRNA clusters show evidence of ping-pong amplification, and are derived from intergenic piRNA clusters. A) Representative 3' UTR cluster, with antisense reads. From top to bottom, the figure shows the annotated 3' UTR (red), the 3' UTR cluster region (blue), and piRNAs (red: plus strand; blue: minus strand). Inset: close-up view of the small region encompassing the 10-bp sense/antisense piRNA overlap typical of ping-pong amplification. B) Annotation of the genomic sources of antisense reads that have 10-bp overlap with sense reads in 3' UTR clusters. Most are derived from intergenic piRNA clusters, but nearly a third come from 3'UTR clusters. To determine the genomic source of the likely piRNAs, we took antisense sequences from locations with evidence of ping-pong, and realigned them to the genome allowing only unique alignments and up to one mismatch (see Methods); this procedure identified the likely genomic origin of 7,295 antisense RNAs that may be primary piRNAs. C) Distribution of the number of sense/antisense overlaps in each region covered by overlapping RNAs. Most sites have only one pair. D) Distribution of 3' UTR cluster coverage by 10-bp sense/antisense piRNA overlaps. The vast majority of clusters contain sense/antisense pairs over less than 10% of their length.

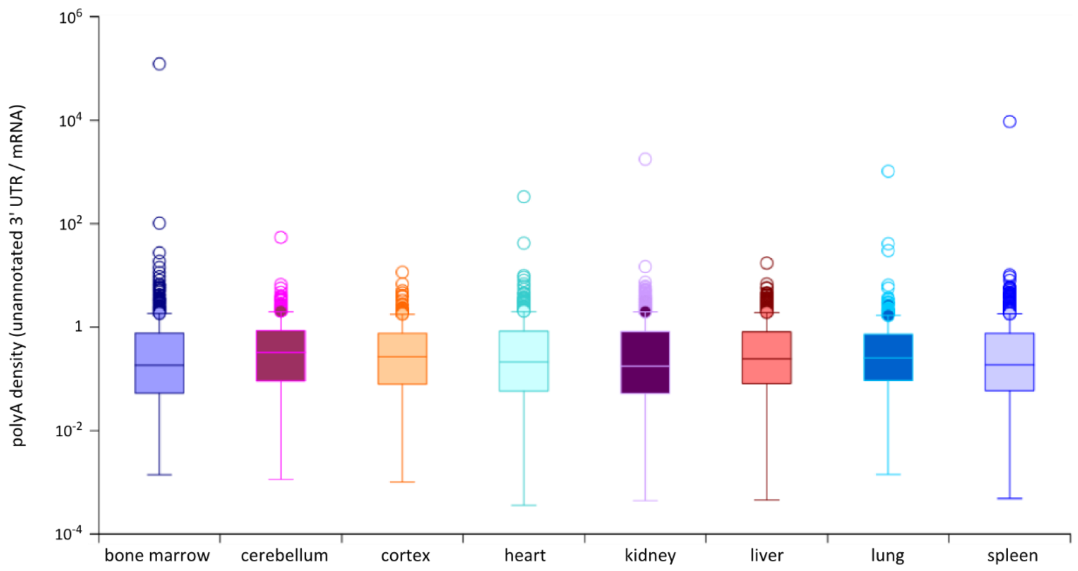

Additional Figure 3: Unannotated 3' UTRs are expressed in somatic tissues. The horizontal axis shows the tissues whose mRNA expression levels were analyzed. The y-axis shows the ratio of expression (read density in RPKM) of the unannotated 3'UTR to expression of the corresponding annotated mRNA: a ratio of 1 indicates equal expression. The extended 3'UTRs are expressed in all tissues: in most cases, the unannotated 3'UTR is less abundant than the canonical annotated mRNA, but in some cases it is more abundant.

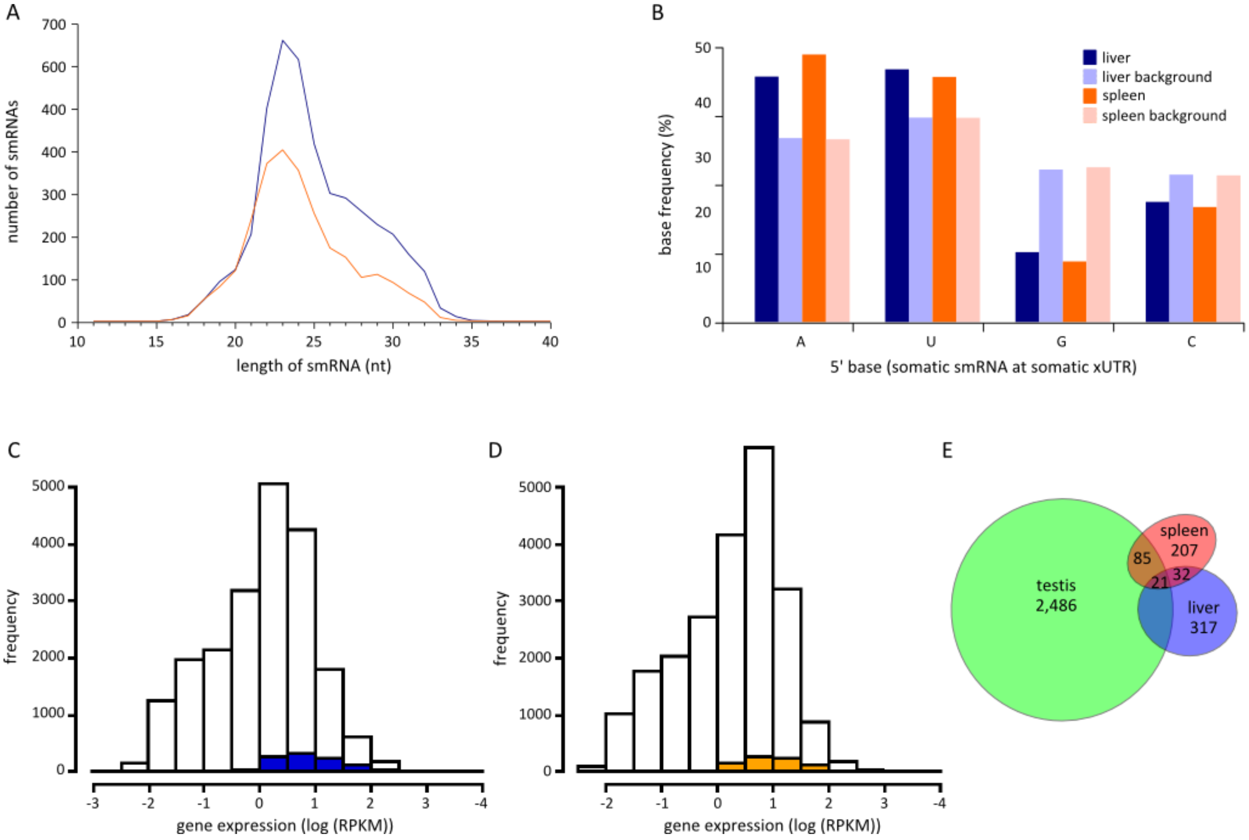

Additional Figure 4: Small RNAs aligning to somatic xUTRs. A) Length distribution of small RNAs from liver (blue) and spleen (red) aligning to somatic xUTRs. **B)** 5' base composition of small RNAS aligning to xUTRs in liver and spleen. C and D) Relationship between gene expression level and the presence of small RNAs aligning to xUTRs in liver **(C)** and spleen **(D)**. Colored bars indicate expression levels of genes with xUTRs; all other genes are white bars. Many highly expressed genes lack 3' UTR small RNA clusters. Expression levels are derived from Encode RNA-seq data. **E)** Venn diagram illustrating the overlap of xUTRs identified in testis, spleen, and liver. Most xUTRs are specific to one of the three tissues.

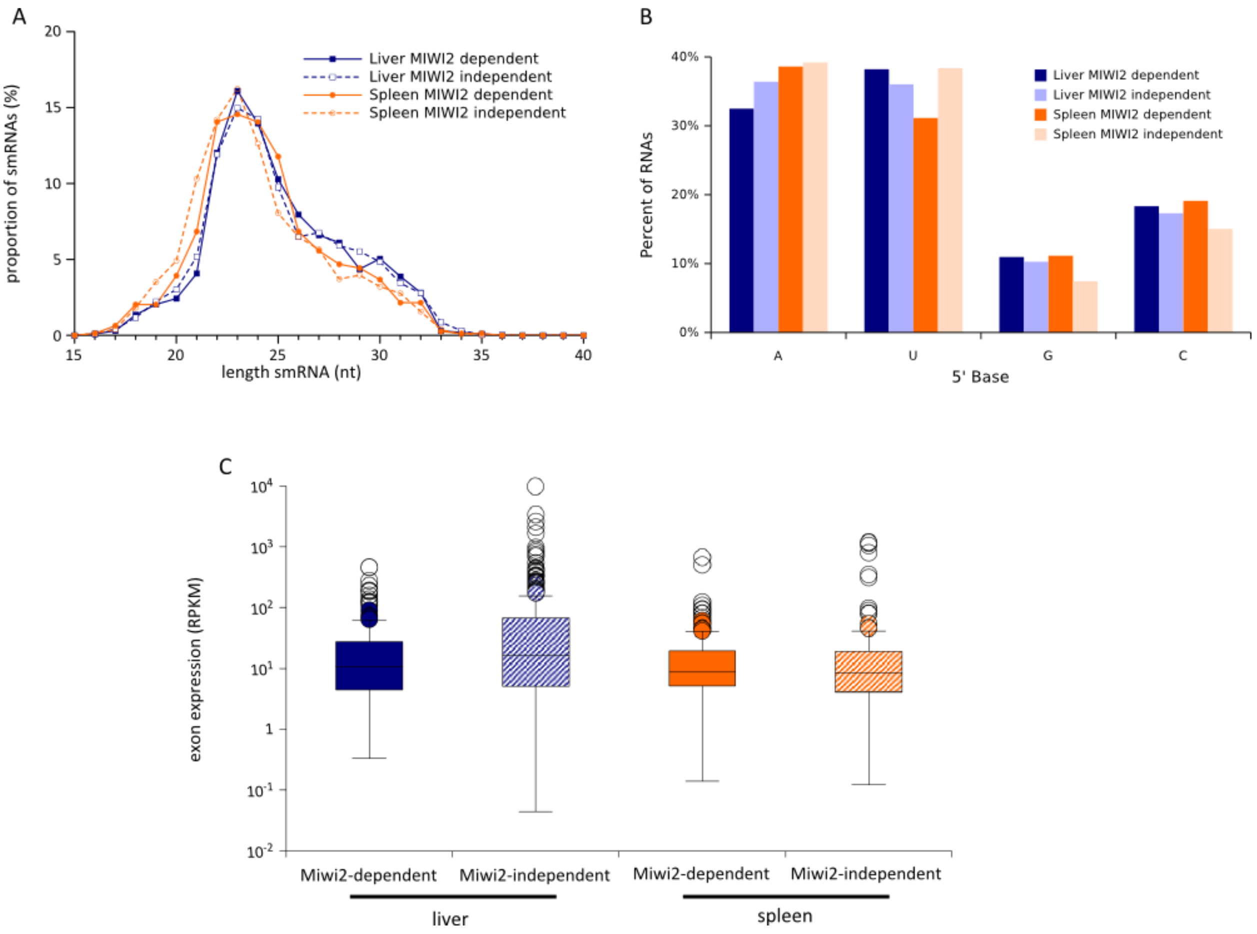

Additional Figure 5: MIWI2-dependent small RNAs aligning to somatic xUTRs. A) Length distribution of MIWI2-dependent and -independent small RNAs in liver and spleen. **B)** 5’ base composition of MIWI2-dependent and -independent small RNAS aligning to xUTRs in liver and spleen. **C)** Expression levels of genes with MIWI2-dependent and -independent somatic xUTR clusters in liver and spleen.

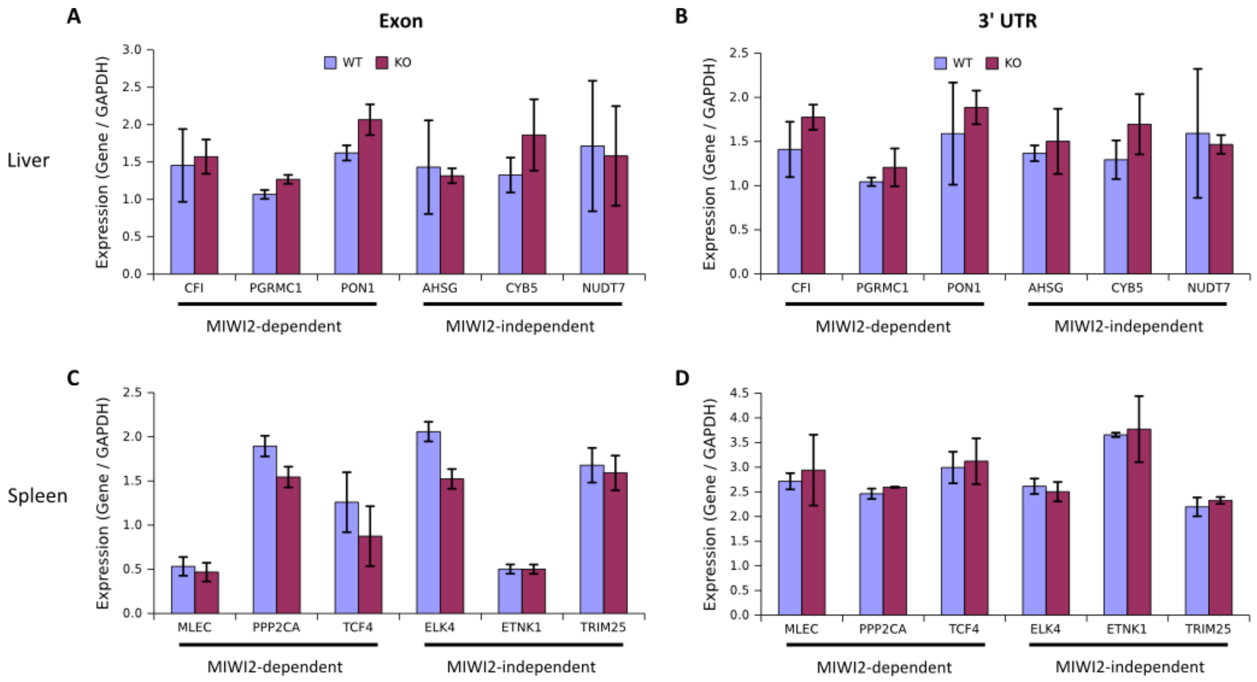

Additional Figure 6: Expression levels of exons and 3'UTRs from genes with MIWI2-dependent somatic xUTR clusters. Expression levels were measured by qPCR of RNA extracted from the liver (A, B) or spleen (C, D) of wild type or MIWI2$^{-/-}$ mice. qPCR was carried out with primers targeting either a coding exon (A, C) or the 3'UTR (B, D). Values shown are the average and standard deviation obtained from two different mice.
